# EMDR and standard psychotherapy for paediatric cancer patients and their families: a pilot study

**DOI:** 10.3389/fpsyg.2024.1407985

**Published:** 2024-07-09

**Authors:** Giulia Zucchetti, Sabrina Ciappina, Elvia Roccia, Deborah Concas, Mario Giordano, Chiara Battaglini, Tiziana Geuna, Claudia Peirolo, Elisa Faretta, Isabel Fernandez, Paola Quarello, Franca Fagioli

**Affiliations:** ^1^Department of Paediatric Onco-Haematology, Regina Margherita Children’s Hospital, AOU Città della Salute e della Scienza, Turin, Italy; ^2^EMDR Italy Association, Bovisio Masciago, Italy; ^3^University of Turin, Turin, Italy

**Keywords:** PTSD, paediatric cancer, EMDR, psychotherapy, paediatric psychology, distress

## Abstract

**Introduction:**

This study examined the efficacy of eye movement desensitisation and reprocessing (EMDR) therapy compared with standard psychotherapy (SP) in treating post-traumatic stress disorder (PTSD) in paediatric oncology patients and their families in the early stage of cancer treatment. The secondary aim of this study was to assess whether EMDR therapy has a different impact on post-traumatic growth compared to SP.

**Methods:**

Forty patients were randomly assigned to EMDR or SP groups. The Impact of Event Scale – Revised (IES-R) and the Distress Thermometer (DT) were used to assess PTSD symptoms at pre-treatment (at cancer diagnosis) and in the post-treatment stages (after 8 sessions). The Post-traumatic Growth Inventory-PTGI was administered in the post-treatment stage in order to evaluate positive changes.

**Results:**

Both EMDR and SP are effective in reducing PTSD, but EMDR was significantly more effective than the SP in reducing scores on the IES-R, especially regarding the intrusive symptom subscale. Also, in the EMDR group there were higher scores of PTGI than in the standard group.

**Conclusion:**

EMDR thus represents a promising treatment in the paediatric psycho-oncology setting.

## 1 Introduction

Research exploring psychological burden among paediatric cancer patients and their families is not new (Lee et al., 2023). However, the classification of this burden as trauma and stressor-related symptoms has been the main focus of research over recent years. Diagnosis of the disease is probably the highest moment of stress for the family as a whole: disruption of life, pain, fear of death, medical procedure, depression and anxiety may occur. If unaddressed, these psychosocial discomforts may develop into high levels of post-traumatic stress symptoms (PTSS) or post-traumatic stress disorder (PTSD). Specifically, some researchers have highlighted that severe distress, indicating trauma, can exist 5–6 weeks after diagnosis (Landolt et al., 2003) and that these post-traumatic stress symptoms may have a protracted course (Lee et al., 2023). Also, subclinical PTSS can continue or manifest during survivorship phases by leading to various negative effects on the quality of life of children and adolescents (Marusak et al., 2019). Among parents of children with cancer, research has shown that they typically experience post-traumatic stress symptoms (PTSS), particularly at the initial stage of the diagnosis (Katz et al., 2018; Carmassi et al., 2021). Symptoms among parents can include, for example, intrusive memories about the moment of the diagnosis and of the child’s treatment (Tremolada et al., 2016). These symptoms can coexist with depression and anxiety, and may have different trajectories in different ethnic groups, as highlighted in a group of parents of children with acute lymphoblastic leukaemia (Chong et al., 2023). Since a cancer diagnosis is a stressful and potentially traumatic experience for children and their families, a screening of possible PTSS and other concurrent stressors is crucial for optimising dedicated psychological intervention to ensure safe mental health throughout treatment and survivorship. However, among children and adolescents, traumatic symptoms can be more difficult to intercept. They can be manifested, for example, through nightmares, sleep disorders or somatic symptoms, and may sometimes be masked by children due to some fears. Thus, screening and intervention should be proposed earlier, in a preventative manner, in order to avoid the onset of severe issues. Eye movement desensitisation and reprocessing (EMDR) can be an appropriate treatment also among children and adolescents (Civilotti et al., 2021), not only for adult cancer patients (Portigliatti Pomeri et al., 2021). Its efficacy has been demonstrated among paediatric patients who have experienced different trauma such as physical violence, psychological disorders and war scenarios. According to the literature, EMDR therapy could be a valid innovative form of care in reducing symptoms also among children and adolescents with physical illness, especially if they require invasive treatment practices and are disabling or chronic conditions (Meentken et al., 2020; Civilotti et al., 2021). Also, a time-limited EMDR has been proven able to reduce PTSD symptoms, psychological comorbidity, and distress in parents of children with a rare progressive life-limiting illness, in particular mucopolysaccharidosis type III (Conijn et al., 2022). No studies have examined its potentiality among children and adolescents with cancer and their families and, to our knowledge, only one case report has described the clinical benefits of the EMDR in an adolescent with cancer (Ciappina et al., 2024). In this case, the standard protocol was altered to focus on the traumatic experience of dealing with cancer (Faretta and Civilotti, 2016). However, given that the results refer to a single case, it is impossible to infer the efficacy of EMDR treatment in paediatric oncology. Thus, the aim of this current research is to evaluate the relative efficacy of EMDR therapy compared with standard psychotherapy (SP) in paediatric oncology patients and parents during the early stage of cancer treatment, as a pilot study. We sought to evaluate the efficacy of EMDR and SP, using a specific instrument for assessing post-traumatic symptoms and based on positive psychosocial outcomes such as positive emotional growth. For this purpose, several self-assessment questionnaires were administered.

## 2 Methods and participants

Forty paediatric oncology patients and parents were recruited from January 2023 to December 2023 from the Department of Paediatric Onco-haematology of the Regina Margherita Children’s Hospital in Turin, one of the main paediatric hospitals in Italy (Zucchetti et al., 2018). The patients have different types of cancer such as leukaemia, lymphoma, bone sarcoma or solid tumours. Patients and parents were offered the chance to participate in the clinical and research psychological protocol EMDR_ITA_PED, approved by the Ethics Committee of AOU Città della Salute e della Scienza of Turin (Prot. No. 0073656; July 2022). Enrolled patients and parents were then randomly assigned to a group receiving EMDR or to a group receiving standard psychotherapy (SP).

Inclusion criteria were: patient just diagnosed with onco-haematological disease; age range ≥ 12 years old; acceptance of informed consent. Exclusion criteria were: patients <12 years old; no acceptance of informed consent. All procedures were performed according to the ethical standards of the institutional and/or national research committee. For both EMDR and SP groups, we proposed 8 sessions of psychotherapy.

**Figure 1:**
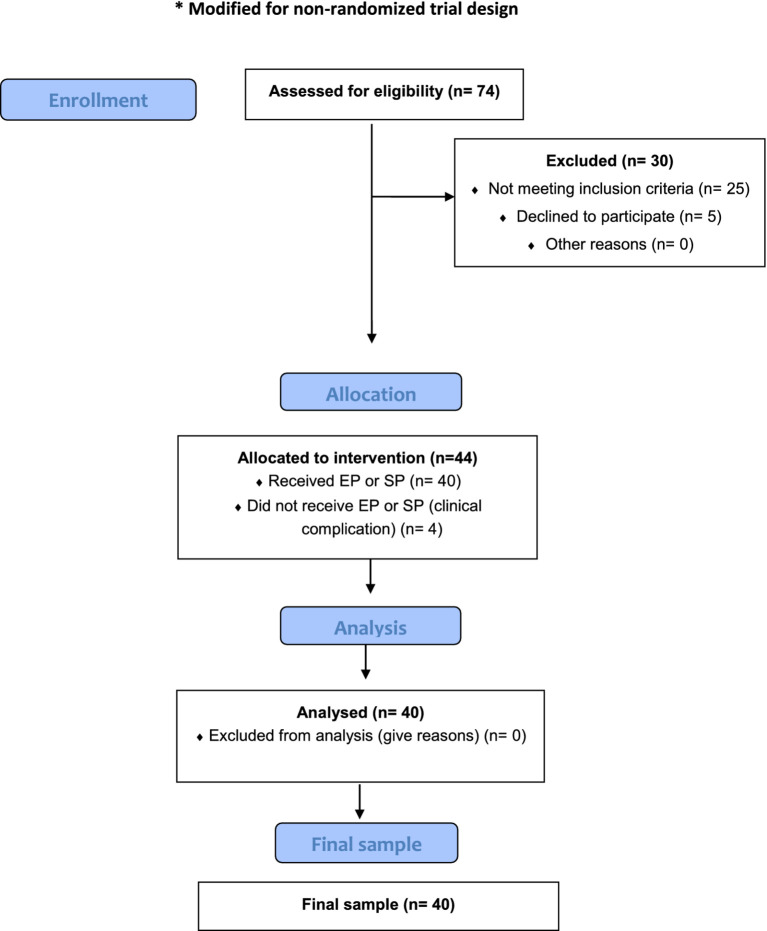
Diagram flow for patients and parents enrollment.

For patients and parents in the EMDR group, we followed the EMDR Protocol proposed by Faretta et al. (2016), a specific protocol for cancer focused on difficulties related to different stages of the illness (Shapiro, 2001; Murray et al., 2010). No pharmacological treatment or other types of psychotherapy were provided in advance for either group. The CONSORT flow diagram for patients and parents’ enrolment, which has been modified for a non-randomised trial, is shown in Figure 1.

### 2.1 Measures

For the first psychotherapy session occurring shortly after the diagnosis communication, patients and parents of both groups were screened simultaneously with a battery of standardised questionnaires to assess PTSD-symptoms: the Impact of Event Scale – Revised (IES-R) (Pietrantonio et al., 2003), the Distress Thermometer (DT) (Grassi et al., 2013), and the Post-traumatic Growth Inventory (PTGI) (Prati and Pietrantoni, 2006). DT and IES-R were administered to patients and parents prior to treatment (in the 1st session), with the same questionnaires and PTGI being administered post-treatment (thus, after 8 sessions). All questionnaires used for the assessment of participants in the study were self-administered under the supervision of the psychotherapist.

Specifically, the Impact of Event Scale – Revised (IES-R) (Weiss, 2007) consists of 22 items answered on a Likert scale from 0 (not at all) to 4 (extremely), designed to investigate post-traumatic symptomatology. It consists of three subscales (intrusion, avoidance, and hyperarousal) that assess subjective distress caused by traumatic events. Respondents are asked to identify a specific stressful life event and then indicate how much they were distressed or bothered during the previous 7 days by each “difficulty” listed. The maximum mean score of each of the 3 subscales is 4, so the maximum total mean score of the IES-R scale is 12. A total IES-R score of 33 or higher out of a maximum score of 88 indicates the likely presence of PTSD, but the cutoff point of 24 indicates partial PTSD or at least some of the symptoms while 33 is the best cutoff for a PTSD diagnosis. The Distress Thermometer was used to assess distress and everyday problems. It consists of a thermometer score measuring overall distress (0 = ‘no distress’ to 10 = ‘extreme distress’), accompanied by a problem list (divided over six domains: practical, family/social, emotional, physical, cognitive, and parenting). Problem domain scores are the sum of the dichotomous items (0 = ‘no’ and 1 = ‘yes’) in each problem domain and a total problem score can be calculated. The Post-Traumatic Growth Inventory (PTGI) (Tedeschi and Calhoun, 1996), is a self-administered questionnaire containing 21 statements concerning personal changes that may occur following a traumatic event. For each statement, the subject must indicate on a grid a response from 0 (no change) to 5 (very important change).

### 2.2 Procedure

#### 2.2.1 SP protocol

SP corresponds to the third level of the Italian protocol of paediatric psycho-oncological intervention (Zucchetti et al., 2020). During the psychotherapy sessions, a psychotherapist offers emotional advice, strategies, and cancer-related knowledge to the parents or patients by helping them activate their personal resources during the cancer experience. Specifically, standard psychotherapy is aimed at sustaining the positive emotional reactions of patients with cancer and of their parents, their adaptive defence mechanisms, the promotion of optional coping strategies, and redirection when defence mechanisms are maladaptive.

#### 2.2.2 EMDR protocol

This protocol was proposed by Faretta et al. (2016) and is used with cancer patients because it is focused on difficulties related to different stages of the illness (Shapiro, 2001; Murray et al., 2010; Ciappina et al., 2024). It was followed for each participant.

- Phase 1: Client history – follows the standard EMDR protocol, with an increased focus on the self/disease relationship and significance of the disease in the patient’s history.
- Phase 2: Preparation – follows the standard EMDR protocol, including time dedicated to psychoeducation on pain and oncological illness.
- Phase 3: Assessment – this is the only phase that differs from the standard EMDR protocol. Targets are related to traumatic experience due to illness, and to concerns and current issues (surgical intervention, treatments, hospitalisation, etc).
- Phase 4: Desensitisation and reprocessing – follows the standard EMDR protocol. In this phase, the role of the therapist as a “safe base” for patients is very important.
- Phase 5: Installation – follows the standard EMDR protocol and integrates the installation of positive cognition.
- Phase 6: Body Scan – is identical to standard EMDR procedure.
- Phase 7: Closing the session – includes the imagery of health resources.
- Phase 8: Re-evaluation – follows the standard EMDR protocol.

### 2.3 Statistical analysis

Data about the possible variable changes from pre-treatment and post-treatment in both groups were analysed using mean values. DELTA values, the mean difference between variables at pre-treatment and post-treatment were also examined. DELTA 1 is the difference between pre-treatment and post-treatment in the EMDR group; DELTA 2 is the difference between pre-treatment and post-treatment in the SP group. An unpaired t-test was used to examine possible significant differences among pre- and post- treatment data.

## 3 Results

There were 40 patients (80% female) and parents (85% mothers) enrolled in the study: 19 of them were in the EMDR group (10 patients and 9 parents) while the other 21 were in the SP group (10 patients and 11 parents). No patients dropped out from the treatment. The numbers of patients and caregivers do not match for each group because not all parents/caregivers were able to complete all of the sessions, either in the SP group or in the EMDR group, for different reasons (particularly for clinical motives such as severe aplasia or severe side effects of the oncological treatment). So, we excluded data not related to the 8 sessions. The mean age of patients was similar in both groups (13.3 for EMDR group and 13.2 for SP group) and also for caregivers (42.1 for EMDR group and 43.4 for SP group). 30 parents (75%) had a lower secondary level of education and 10 parents (25%) had upper secondary level schooling. Considering employment levels, 28 parents (70%) were white collar employees, 5 parents (12%) were blue collar employees and 7 parents (8%) were middle managers. There were no particular differences in clinical variables between the two groups at baseline (see Table 1). As we noted, the mean value for the total of IES-R at the pre-treatment stage was high in both groups (48 in the EMDR group and 47 in the SP group) which denoted a presence of PTSD immediately after the communication of diagnosis. Also, the means of the 3 subscale scores were high in both groups. A high value was recorded at the pre-treatment stage in both groups regarding distress symptoms (EMDR group 8.7 vs. SP group 8.7). We evaluated whether the different psychotherapy treatments (EMDR or SP) administered to patients and parents had a different impact on the psychological variables involved. After the EMDR therapy and SP, these values went down but a greater decrease between pre-treatment and post- treatment can be noted in the EMDR group, especially concerning the post-traumatic symptoms examined through the IES-R. Specifically, the IES-R total score in the EMDR group decreased until it is no longer frankly pathological (EMDR group pre-treatment 48 vs. EMDR group post-treatment 24). This decrease is particularly clear in the value of intrusion scale (EMDR group pretreatment 3.6 vs. EMDR group post-treatment 1.7). The DELTA values in Table 2 confirm the highest reduction of the psychological variables in the EMDR group, especially for the total score (24 in EMDR group vs. 11 in SP group; *t* = 2.5, *p* < 0.05) and for the intrusion scale (1.9 EMDR group vs. 1.2 in SP group; *t* = 1.9, *p* < 0.05). In the other subscales, a t-test did not show statistically significant differences between pre- and post- treatment.

**Table 1:** Means scores from pre-treatment to post-treatment among EMDR and SP groups.

	Pre-treatment		Post-treatment	
	EMDR group *N* = 19	SP group *N* = 21	EMDR group *N* = 19	SP *N* = 21
IES – R total	48 (17.2)	47 (16.1)	24 (9.4)	36 (12)
*Intrusion*	3.6 (9.8)	3.5 (9.2)	1.7 (6.3)	2.1 (7.4)
*Avoidance*	3.4 (7.3)	3.3 (7.1)	1.9 (3.8)	2.3 (5.7)
*Hyperarousal*	3 (8.5)	3 (6.9)	1.9 (5.8)	2.1 (7)
DISTRESS TEST	8.7	8.7	4.5	4.8
PTGI total				
*Personal strength*	/	/	5	4
*Relating to others*	/	/	3	2
*New possibilities*	/	/	5	3
*Appreciation of life*	/	/	2	3
*Spiritual change*	/	/	3	2

Subscales data are mean. IES-R, Impact of Event Scale-Revised; PTGI, Post-traumatic Growth Inventory.

**Table 2:** Delta values in the IES-R scores (pre-treatment – post-treatment) among groups.

	EMDR group	SP group
	Δ pre-post	Δ pre-post
IES-R total	24	11
*Intrusion*	1.9	1.2
*Avoidance*	1.5	1
*Hyperarousal*	1.1	0.9

Δ = mean difference between variables at pre-treatment and post-treatment.

## 4 Discussion

The primary aim of this study was to evaluate the relative efficacy of EMDR therapy compared with SP in paediatric oncology patients and their parents after the cancer diagnosis. A child or adolescent cancer diagnosis is a stressful and potentially traumatic experience for the family as a whole, so an earlier intervention is necessary in order to avoid potential post-traumatic symptoms. To our knowledge, no studies have evaluated the efficacy of EMDR therapy on this type of population and none have compared EMDR to standard psychotherapy on specific measures of post-traumatic symptoms and on positive psychosocial outcomes such as positive emotional growth. First of all, our results underline that, immediately after cancer diagnosis, cancer patients and their caregivers suffer from significant post-traumatic symptoms such as reaching a risk value for PTSD. Patients and parents reported intense emotional activation leading to fear, intrusive thoughts, flashbacks and nightmares. Also, they demonstrated general distress, especially in carrying out daily life activities. The most significant result emerging from this study is that most patients and parents treated with EMDR were able to significantly reduce their symptoms of post-traumatic stress. Although this result is shown also in the group of patients who are treated with standard psychotherapy, results showed that the decrease of all symptoms is more evident in the EMDR group. Also, the group treated with EMDR had lower IE-R total scores, with intrusive, avoidance and hyperarousal symptoms after the psychological treatment, as compared with the group of participants treated with SP. These results are in line with the literature about the efficacy of EMDR with oncological adult patients, confirming that EMDR is a more effective and rapid therapy, in particular for reducing stress symptoms such as the intrusive feelings that are typical in the oncology field (Capezzani et al., 2013; Faretta et al., 2016).

Other important findings highlighted that participants feel stronger and seem to glimpse new possibilities; these results are especially high in the EMDR group. This could be linked to recent studies which have suggested that the cancer experience can also lead to positive outcomes, including PTGI (Arpawong et al., 2013). The concept of PTGI can be defined as a positive psychological change, experienced as a result of a struggle with highly challenging life circumstances (Yi and Kim, 2014). In this case, although a diagnosis of cancer in childhood or in adolescence is a traumatic experience, if treated through EMDR therapy as soon as possible after the diagnosis, it could reduce the onset of PTSD symptoms. Future studies should confirm this hypothesis. This current study has several limitations. The number of included patients and parents treated with EMDR and SP is not large and this may limit the generalisability of the findings to all paediatric cancer patients; our future goal is to increase the sample size in order to examine both demographic and clinical (tumour histology) possible differences by reducing potential bias. Future studies should also take into account age differences regarding cognition and emotion regulation strategies that could influence the effects of the technique, and could be designed to examine any differences between patients and parents; they should also include siblings. Also, a future study should provide for a follow-up of at least 6 months after the end of treatment, to prove the stability of the treatment effects. To bypass the methodology limitations, further studies should incorporate a mix of qualitative and quantitative methods (such as semi structured interview) to provide a more comprehensive understanding of the therapies’ impact. For this pilot study, our working group agreed on self-administered tests. However, these were always submitted to the participants during the session and filled in under the supervision of the psychotherapist. From our qualitative point of view, we have not observed to date any difference between our observations and what is reported by the patients.

Although our results can only be considered preliminary, this pilot study suggests that, in paediatric cancer patients and parents, EMDR had an advantage over SP in decreasing post-traumatic symptoms. Our study is in line with the most recent literature that suggests EMDR as an effective therapy for oncological patients who have received a cancer diagnosis, and it is innovative in the field of paediatric oncology (Gainer et al., 2020; Abdi et al., 2021; Portigliatti Pomeri et al., 2021; Lee et al., 2023). EMDR therapy can help in quickly reducing post-traumatic symptoms from which they suffer after the terrible news of oncological disease. So, it is crucial that paediatric patients and parents have access to a post-traumatic symptoms screening in order to obtain dedicated psychological support such as EMDR, which has been shown to be effective in helping patients and parents to cope with initial stress symptoms and find the strength to face the new situation. Our data firstly contributes to the knowledge about use of EMDR in the paediatric oncology field and this study opens the door to future research with this type of fragile population.

## 5 Conclusion

To conclude, our study suggests that both EMDR and SP are effective in treating many stress symptoms in paediatric oncology patients and parents, but our results suggest that EMDR could be a more effective therapy for this population with a high level of post-traumatic symptoms, in particular intrusive symptoms and especially during the early active stage of treatment.

## Data availability statement

The raw data supporting the conclusions of this article will be made available by the authors, without undue reservation.

## Ethics statement

The studies involving humans were approved by AOU città della salute e della scienza torino. The studies were conducted in accordance with the local legislation and institutional requirements. Written informed consent for participation in this study was provided by the participants’ legal guardians/next of kin.

## Author contributions

GZ: Writing – original draft, Writing – review & editing. SC: Writing – original draft, Writing – review & editing. ER: Writing – review & editing. DC: Writing – review & editing. MG: Writing – review & editing. CB: Writing – review & editing. TG: Writing – review & editing. CP: Validation, Writing – review & editing. EF: Conceptualization, Methodology, Supervision, Writing – original draft, Writing – review & editing. IF: Conceptualization, Investigation, Methodology, Supervision, Validation, Writing – original draft, Writing – review & editing. PQ: Writing – review & editing. FF: Writing – original draft, Writing – review & editing.
